# Effective control of sickle cell disease with hydroxyurea therapy

**DOI:** 10.4103/0253-7613.62409

**Published:** 2010-02

**Authors:** Harminder Singh, Navin Dulhani, Bithika Nel Kumar, Prabhakar Singh, Pawan Tiwari

**Affiliations:** Department of Pharmacology, Government Medical College, Jagdalpur, Chhattisgarh, India

**Keywords:** Fetal hemoglobin, Sickle cell anemia, Hydroxyurea, Sickle cell disease

## Abstract

**Objective:**

Hemoglobin F augmentation is another approach to treat sickle cell disease (SCD). This study evaluates the efficacy and impact of Hydroxyurea (HU) on fetal hemoglobin (HbF) and other hematological parameters, which result in decreasing the painful crisis and lower hospital admissions.

**Materials and Methods:**

A prospective study was carried out in the Department of Medicine, Government Medical College, Jagdalpur. Twenty-seven patients with SCD received HU at a mean dose of 22 mg/kg/d. The baseline results were analyzed and compared with the post treatment result, at the end of one year.

**Statistics:**

Student's t-test was used to determine the level of significance.

**Results:**

Twenty-four patients completed a one-year period successfully; a significant increase was noted in the mean HbF%, from 12.83 to 19.17, and the mean corpuscular volume (MCV) from 82.57 to 89.87 Fl. The mean hospital admission (numbers) in the last one year decreased from 4.75 to 2.25 and the mean number of SCD crisis for the last one year decreased significantly from 3.63 to 1.67.

**Conclusion:**

We found a significant reduction in hospital admissions, a reduction in the overall sickle cell crisis and an associated improvement in HbF% without any significant side effects in the patients with SCD, treated with HU.

## Introduction

More than 50 years ago Janet Watson recognized that the red cells of infants with sickle cell trait failed to sickle *in vitro*; she hypothesized that these observations were due to the elevated HbF levels in infant blood. HbF interfered with HbS polymerization and it was appreciated that enough HbF, if evenly distributed among sickle erythrocytes, might ‘cure’ the sickle cell disease. A search was launched for pharmacological agents that could reverse the switch from gama to beta globin chain synthesis.[1] Bone marrow transplantation (BMT) was the only curative therapy, but its use was often limited by the lack of an HLA-compatible healthy donor. Reactivation of fetal hemoglobin (HbF) synthesis may be an alternative approach; a number of trials are now being conducted to test this hypothesis.[2,3]

Beta globin chains characterize HbF and sickle gama globin chains are present in HbS. The first ‘hemoglobin switching’ agent, a nucleoside analog 5-azacytidine, was postulated to increase HbF by inducing gene expression. The other drug, Hydroxyurea (HU), is the prototype known to promote HbF production indirectly by perturbing the maturation of erythroid precursors, is administered orally with a once-daily dosing, has no immediate adverse effects, and is generally effective for patients with sickle cell disease (SCD).[4,5]

The multicenter Phase I / II safety trial of HU therapy for severely affected school-aged children with SCA (HUG KIDS) demonstrated significant increases in hemoglobin concentration, mean corpuscular volume, and the percentage of HbF.[6] Subsequently, HU has been shown to aid the growth and development of children with SCD and prevent stroke recurrence in children with previous cerebrovascular accidents.[7]

An open-label study of HU in 32 patients with SCD showed that HbF synthesis increased in most patients treated with doses of HU, which produced limited myelotoxicity. HbF responses were dose-related, and in some patients the levels were as high as 15 to 20%. Laboratory studies suggested that HbF levels of at least 15 to 20% might be required for a clinical benefit, but in a study of untreated patients, it appeared that any increase above 4% might be beneficial.[8,9]

Clinical efficacy has also been proven; in a double-blinded, placebo-controlled, randomized trial, involving severely affected adults with SCD, HU significantly reduced the number of painful vaso-occlusive events, blood transfusions, episodes of acute chest syndrome, and hospitalizations. More recently, long-term HU use has been shown to decrease mortality in adults with SCD.[10] For pediatric patients, HU has a similar toxicity profile, with only mild, transient, and reversible myelosuppression.[11] For adult patients, hydroxyurea has dose-related hematological efficacy and an acceptable short-term toxicity profile.[12,13]

Recent findings suggest that the induction of HbF by HU involves the activation of soluble guanylate cyclase by NO, and reduces the rates of vaso-occlusive crisis in patients with SCD and recent data suggest that HU treatment can generate nitric oxide (NO). Nitric oxide has been proposed as a novel therapy for sickle cell disease via a number of pathways.[14,15]

In a backward State like Chhattisgarh where the prevalence is about 22% for the sickle cell trait and 2 – 3% for full-blown sickle cell disease, where blood is in short supply, blood banking facilities are primitive, and voluntary blood donation is not a common practice, management of this disease becomes difficult.[16] Owing to social and economic reasons, patients cannot maintain the ideal pre-transfusion hemoglobin of 9 to 10.5 g/dL. In such resource-restricted settings, HU becomes the best alternative. The main objective of our study therefore is to evaluate the efficacy and safety of HU in SCD patients.

## Materials and Methods

The study was conducted from March 2008 to March 2009, in the Department of Medicine with help from the Pharmacology Department, Government Medical College, Jagdalpur, Chhattisgarh, India. Twenty-seven subjects were included, after obtaining informed consent and approval from the Institutional Ethics Committee.

### Inclusion and exclusion criteria

All patients who had homozygous SCD proven by Hb electrophoresis, with a history of severe, recurrent vaso-occlusive sickle cell pain that required three or more hospitalizations per year, stroke or acute chest syndrome, and severe or symptomatic anemia (Hb < 7 g/dL) were included in the study.

Patients who were pregnant or who were sexually active and unwilling to use contraception, those with active liver disease (HBV or HCV infection), those previously treated with HU or other anti-sickling agents, and patients with a history of significant non-compliance with recommended medical care, were excluded.

Patients who satisfied the inclusion and exclusion criteria were examined for baseline demographic data. Baseline investigations were carried out, including Hb electrophoresis, with quantitative HbF%, CBC with differential and reticulocyte count, biochemistry profile (electrolytes, LDH, total protein, albumin, total bilirubin), liver function tests (AST, ALT), renal function tests (BUN, Cr), serum B-12 and folate (to ensure HU-related macrocytosis does not mask deficiency), serum iron, total iron binding capacity, ferritin (to ensure that HU related-macrocytosis does not mask Fe-deficiency), and pregnancy test for females.

Follow-up visits were scheduled every two months. On each visit, clinical and laboratory examination including weight, blood pressure, vital signs, toxicity focused physical examination, and inquiry regarding side effects, sexual activity, and birth control, pulse saturation of oxygen (Sa02), and complete blood count (CBC) with differential and reticulocyte count were performed. Quantitative HbF%, ALT, AST, BUN, and Cr were repeated every six months.

The main evaluation was limited to Hb level, HbF, MCV, mean corpuscular hemoglobin concentration (MCHC), and WBC count at the end of one year of treatment with HU. Values within three months after the last transfusion were not considered. The percentage of HbF was determined by scanning after citrate agar gel electrophoresis, in the Pathology Department: The data of the previous one year, related to the number of crises and number of hospitalizations from the patients’ discharge slips were recorded and the same data were recorded after one year of hydroxyurea treatment.

The initial dose of HU was 20 mg/kg daily; it was increased by 5 mg/kg if judged appropriate by the treating clinician. Treatment attitude was mainly based on the clinical response of each patient at the starting dose of 20 mg/kg. There were no fixed guidelines for dose escalation and dose modification as long as the patient was found to be adequately controlled by the treating physician. There was never any attempt to reach a ‘maximal tolerated dose.’ All SCD patients on HU were seen every two months for routine evaluation and screening for any hematological toxicity.

### Statistical analysis

The values were expressed as a mean and 95% confidence interval of the mean; the paired Student's t-test was used to determine the level of significance of differences between the biological parameters, before and after HU treatment. *P* < 0.05 was considered to be significant.

## Results

Twenty-seven patients were enrolled in the study. Three patients were excluded after four to five months as they failed to attend the scheduled evaluation visits of the second follow up. There were thus 24 patients whose results could be analyzed. The demographic data of these patients are indicated in Table 1.

**Table 1:** Demographic data of patients

*Variables*	*n = 24*
Mean age in years	19.85
CI	18.59 – 21.11
Male %	13 (54.16%)
Female %	11 (45.83%)
Mean weight in Kg.	51.96
CI	51.43 – 52.49

CI = 95%Confidence interval of mean

The mean initial hemoglobin level was 9.15 g/dL. After one year of HU treatment, the mean hemoglobin level increased to 9.98 g/dL.The difference was not significant (*P* = 0.25). The mean initial MCV was 82.57 fL. After one year of HU therapy, it increased to 89.87fL. This change was highly significant (*P* < 0.001; Table 2). The MCHC did not change significantly (*P* = 0.36).

**Table 2:** Effect of hydroxyurea on the hematological profile of patients

*Parameters Mean/CI*	*Before treatment (BT) n = 24*	*After treatment (AT) n =24*	*P*
Mean Hb (g %)	9.15	9.98	0.253
CI	8.55 – 9.75	9.42 – 10.54	
Mean MCV(FL)	82.57	89.87	(0.0045)
CI	80.53 – 84.61	87.52 – 92.22	(significant)
Mean MCHC	31.08	31.46	0.36
CI	30.6 – 31.56	31.05 – 31.87	
Mean WBC	9.62	8.33	0.177
CI	8.87 – 10.37	7.89 – 8.77	
Mean HbF%	12.83	19.17	(0.01)
CI	11.04 – 14.62	17.38 – 20.96	(significant)

CI = 95%Confidence interval of mean, BT = Before treatment, AT = After treatment.

The mean initial HbF value was 12.83%; after one year of HU therapy, it increased to 19.17%. This difference was significant (*P* < 0.05). A threefold increase of the initial HbF value was observed in 11 patients; a twofold increase was observed in seven patients. The increase of HbF correlated significantly with the increase of MCV. In some patients, the MCV increased weeks before any increase in the HbF level. The baseline WBC count was 9.62 (×109 L); after one year of HU treatment, it lowered non-significantly to 8.33(×109 L).

There was statistically relevant change observed in the yearly crisis rate and the number of hospital admissions in the last one year, as shown in Table 3.

**Table 3:** Effect of hydroxyurea on the number of hospital admissions and the crisis in the last one year

*Parameters Mean/CI*	*Before treatment(BT) n = 24*	*After treatment(AT) n = 24*	*P BT × AT*
Mean no. of hospital admissions(in the last one year)	4.75	2.25	0.03
CI	4.19 – 5.31	1.87 – 2.63	(significant)
Mean no. of crisis(in the last one year)	3.63	1.67	0.008
CI	3.32 – 3.94	1.39 – 1.95	(significant)

CI = 95% Confidence interval of mean, BT = Before treatment, AT = After treatment.

The primary toxicity observed in the patients was mild, reversible bone marrow suppression, but the thresholds for toxicity occurred in approximately 5% of the blood counts (data not shown). Usually mild neutropenia was observed, which recovered within one week of temporary discontinuation of HU treatment.

## Discussion

A significant increase in HbF% from 12.83 to 19.17% after one year treatment with HU, was observed. Simultaneously, a significant rise of MCV (from 82.57 fL to 89.87 fL) was also noted. We also noted a positive correlation between HbF% and rise in MCV in patients taking HU for the last one year.

In another study by Ferster *et al.*, similar results were reported, but they did not report lowering of the annual sickle cell crisis.[17] We found a significant decrease in the number of sickle cell crisis and a marked lowering of annual hospital admissions. In a similar study by Ferster *et al.*, with five years of treatment with HU, the results were similar to our study, but significant results were obtained only after a period of two years.[18,19]

Allogenic BMT remains the only curative therapy in severe SCD,[19,20] but in case of patients lacking an HLA-identical sibling, refusing the BMT procedure, or those too old for BMT, or in whom other contraindications exist; HU is the only drug that has proven to modify the disease at short- or middle-term with acceptable toxicity.

HU increases the fetal hemoglobin production in SCD. It promotes fetal hemoglobin production via reactivation of the gamma-globin gene. A series of clinical trials with HU for SCD proved that this medicine is effective and significantly reduces painful crises, occurrence of chest syndrome, and the frequency of transfusions.[21,22]

In our experience, HU therapy was safe with no clinically significant hematopoietic depression requiring cessation of drug therapy. Most of the patients were maintained on doses of 20 to 25 mg/kg per day throughout the entire follow-up period, without any evidence of loss of efficacy, as assessed by the hematological parameters, annual hospitalization rate, and crisis rate. A study by Zamani *et al.*, reported a well-tolerated five-year HU therapy with only transient thrombocytopenia and Steinberg *et al.,* also reported the same result with a nine-year HU therapy.[10,23] Contrary to this a case report by Issaivanan *et al.,* reported a 10-year-old boy with chronic myelogenous leukemia who presented with hyperpigmentation of the skin and nails three months after the start of hydroxyurea therapy.[24]

Compliance with HU therapy is important for achieving a sustained hematological effect in patients with SCD, and this requires a coordinated effort by the medical team and frequent contact with patients and families to provide support and encouragement.

The potential for using long-term HU therapy to reduce the morbidity and mortality of children with SCD requires additional and careful investigation, but HU currently provides the best available strategy to achieve hematological and clinical improvement of the disease.

## Conclusion

We found a significant reduction in hospital admissions, increased intervals between transfusions, reduction in overall sickle cell crisis, and associated improvement in HbF% in SCD treated with HU. The outcome of the present study and the available evidences recommend a wider adoption of HU for treatment in high-prevalence areas.
